# Supplementary material for: Transcriptome and metabolome analyses revealed that narrowband 280 and 310 nm UV-B induce distinctive responses in Arabidopsis
Source: Sci Rep. 2022 Mar 12;12:4319. doi: 10.1038/s41598-022-08331-9 (PMC8918342; doi:10.1038/s41598-022-08331-9)
Supplement: Supplementary file 1 — Supplementary Figures. [file 41598_2022_8331_MOESM1_ESM.pdf]

Transcriptome and metabolome analyses revealed that narrowband 280 and 310 nm UV-B induce distinctive responses in Arabidopsis

Tomohiro Tsurumoto, Yasuo Fujikawa, Yushi Onoda, Yukari Ochi, Daisaku Ohta and Atsushi Okazawa

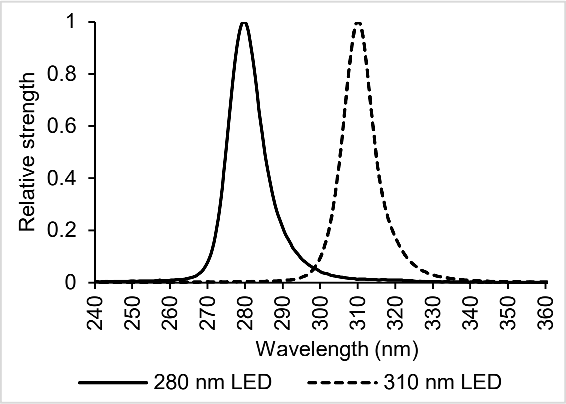

Supplemental Figure S1. Relative emission spectra of 280 and 310 nm UV light emitting diodes (LEDs) used in the experiments.

The 280 nm UV-LED has a peak wavelength of 280 nm, a half-width of 10 nm, and a range from 260 to 310 nm. The 310 nm UV-LED has a peak wavelength of 310 nm, a half-width of 10 nm, and a range from 290 to 340 nm.

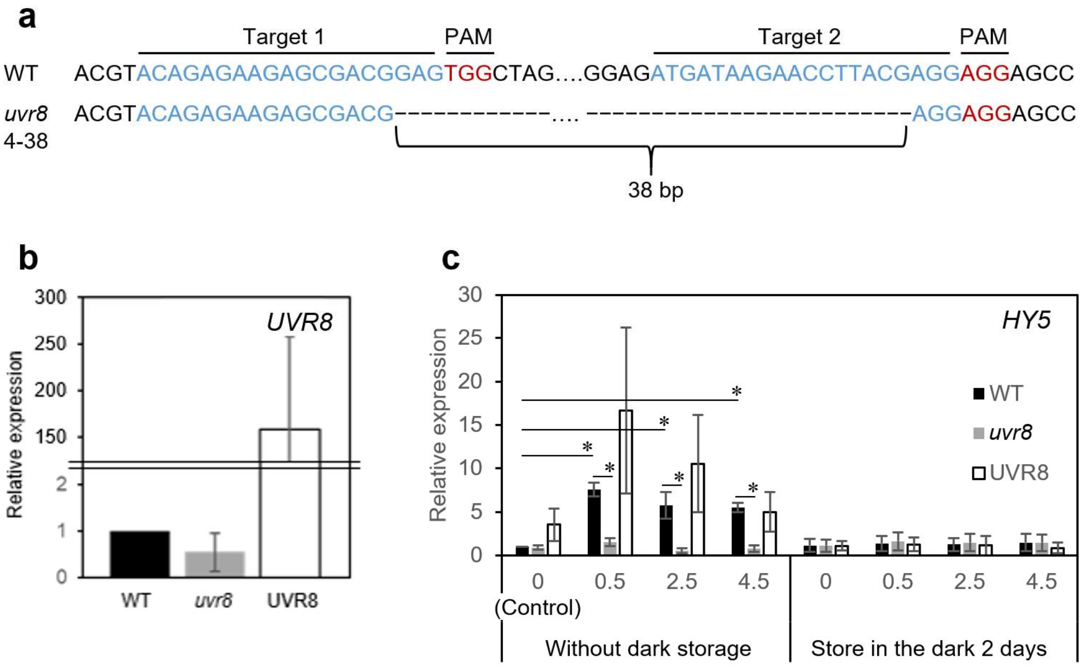

d

AT5G11260 :	CGGGAATCGAAAACGCAACCTTTCTACTACAGTGTCACAATGTCATATCATAGAACAAAAATTAGCTTATTTTTGTTATAAAACAACATT
uvr8 :	CGGGAATCGAAAACGCAACCTTTCTACTACAGTGTCACAATGTCATATCATAGAACAAAAATTAGCTTATTTTTGTTATAAAACAACATT
	ATGTTTCAATTAAGAGAAATCTAAGACTACAATAAGAGAACTTGGTCTACAATTGCAATATTAGCTCTCACATCCCATATAATTTTAACA
	ATGTTTCAATTAAGAGAAATCTAAGACTACAATAAGAGAACTTGGTCTACAATTGCAATATTAGCTCTCACATCCCATATAATTTTAACA
	TGATAATTATTGATACAATTCTCTGTAAATTTTATCCACAAAAATATAACACAAGAAGAAGAAGGAGATCAAAGGCTTGCATCAGCATTA
	TGATAATTATTGATACAATTCTCTGTAAATTTTATCCACAAAAATATAACACAAGAAGAAGAAGGAGATCAAAGGCTTGCATCAGCATTA
	GAACCACCACCACCTCCTCTCTTGTTTCCTGTTGTGTTCTTCAGAATCTACACCACATAAAAAACATAACAACTCAAAAGACTTTATTAC
	GAACCACCACCACCTCCTCTCTTGTTTCCTGTTGTGTTCTTCAGAATCTACACCACATAAAAAACATAACAACTCAAAAGACTTTATTAC
	CACACACACACATAGAGATCCAACTTTGCAATCTCATCTTCTCCATTCATATAGAACAAAATGAGTGAGCATTTCAAGAACCATTGAAGA
	CACACACACACATAGAGATCCAACTTTGCAATCTCATCTTCTCCATTCATATAGAACAAAATGAGTGAGCATTTCAAGAACCATTGAAGA
	ATTTACATGCCTTTTGAGAGAATATGCGAGTGAATGACCATTTCAAGAACCTACATGCCTTCTGAGAATTAATCTAAAGCTTAAGTTAGC
	ATTTACATGCCTTTTGAGAGAATATGCGAGTGAATGACCATTTCAAGAACCTACATGCCTTCTGAGAATTAATCTAAAGCTTAAGTTAGC
	TTCTTAGATCCTTTTAACTAACTAAACTAATTATTGGTCAATCCTAGACTCGTAAATGTGATAAACCAGTACTGTGATATATCAAAAAAC
	TTCTTAGATCCTTTTAACTAACTAAACTAATTATTGGTCAATCCTAGACTCGTAAATGTGATAAACCAGTACTGTGATATATCAAAAAAC
	AAATGGCAAAAGCATTGACGTTGCAGGTTAAGTCAACAGTAAGATCGACAAAACGTACATGTCTAAGCATCTGGTTCTCGTTCTGAAGAG
	AAATGGCAAAAGCATTGACGTTGCAGGTTAAGTCAACAGTAAGATCGACAAAACGTACATGTCTAAGCATCTGGTTCTCGTTCTGAAGAG
	TAGAGAGTCGCTCTTCAAGTTCAGAGTTTTTGTTCTCCAAGTCTTTCACTCTGTTTTCCAACTCGCTCAAGTAAGCCTTTTTCCTCTCTC
	TAGAGAGTCGCTCTTCAAGTTCAGAGTTTTTGTTCTCCAAGTCTTTCACTCTGTTTTCCAACTCGCTCAAGTAAGCCTTTTTCCTCTCTC
	TTGCTTGCTGAGCTGAAACTCTGTTCCTCAACAACCTTTTCACCACAAAATTACCAAACAACCCCATCACGCAACCGTTATTTAACATAA
	TTGCTTGCTGAGCTGAAACTCTGTTCCTCAACAACCTTTTCACCACAAAATTACCAAACAACCCCATCACGCAACCGTTATTTAACATAA
	TCACCTTCCATATAAAGGGTAAAAATGTAAATTCAATGAATAGAGAAAAAGACACCTCTTCAGCCGCTTGTTCTCTTTCTCCGCCGGTGT
	TCACCTTCCATATAAAGGGTAAAAATGTAAATTCAATGAATAGAGAAAAAGACACCTCTTCAGCCGCTTGTTCTCTTTCTCCGCCGGTGT
	CCTCCCTCGCTTCCTTTGACTTTCTCCGACAGTCGCCTGTGTCCGCTCCTGACCGGTCGCCGATCCAGATTCTCTACCGGAAGTTTCTTT
	CCTCCCTCGCTTCCTTTGACTTTCTCCGACAGTCGCCTGTGTCCGCTCCTGACCGGTCGCCGATCCAGATTCTCTACCGGAAGTTTCTTT
	TCCGACAGCTTCTCCTCCAAACTCCGGCACTCGCCGTATCTCCTCATCGCTTTCAATTCCTTTAAAACATAAAAGAGACTTTAGACGAAA
	TCCGACAGCTTCTCCTCCAAACTCCGGCACTCGCCGTATCTCCTCATCGCTTTCAATTCCTTTAAAACATAAAAGAGACTTTAGACGAAA

AGTTTCAAACTTTTTAAATACAATAAAAAATTGCAGATCTTCTGGGGGAGACTAAAAGTTGTGAATCTAGATGTGAATCAATGGTGATAC
AGTTTCAAACTTTTTAAATACAATAAAAAATTGCAGATCTTCTGGGGGAGACTAAAAGTTGTGAATCTAGATGTGAATCAATGGTGATAC

AAAATCTAGATGTGAATTTACTAGATATCCAATGCATGAGAATGAAAATCAATGAGATCACTCGTTGGGAGAAGATATGAAAATAAAACA
AAAATCTAGATGTGAATTTACTAGATATCCAATGCATGAGAATGAAAATCAATGAGATCACTCGTTGGGAGAAGATATGAAAATAAAACA

ATCGACAATTTTTGTTTACCTTCTTTGATCTCCAAATGTGGAGCAGAGCTTGATGACCTCTCGCTGCTTGATGGTAAAGAGCTTGCAGCT
ATCGACAATTTTTGTTTACCTTCTTTGATCTCCAAATGTGGAGCAGAGCTTGATGACCTCTCGCTGCTTGATGGTAAAGAGCTTGCAGCT

AAAGAGCTAGTCGCTTGTTCCTGCATTTTTCTTACTCTTTGAAGATCGATCAGGCGAGAGAGAGAGGGAAAGATTTGTTGTCTTAGTAGC
AAAGAGCTAGTCGCTTGTTCCTGCATTTTTCTTACTCTTTGAAGATCGATCAGGCGAGAGAGAGAGGGAAAGATTTGTTGTCTTAGTAGC

GAAGCTGGTGAAAAGGATAAAGAGGAAAAGAGCGGGACTTGGGATTTACAGAAGAACTTGGAAAGAATCTAATTGCGAGACATTTTGGGA
GAAGCTGGTGAAAAGGATAAAGAGGAAAAGAGCGGGACTTGGGATTTACAGAAGAACTTGGAAAGAATCTAATTGCGAGACATTTTGGGA

AGGAATAGATTACTCTGGCTACCGCCGTCAGATCTCTGAAGAATCGAACGGATATCGAGAGTGAATCGTGGAGAAAGGTGAGCCTTGAGG
AGGAATAGATTACTCTGGCTACCGCCGTCAGATCTCTGAAGAATCGAACGGATATCGAGAGTGAATCGTGGAGAAAGGTGAGCCTTGAGG

AAATACAAGGATCCAAAGGCAATTGAGAT
AAATACAAGGATCCAAAGGCAATTGAGAT

Supplemental Figure S2. Characteristics of transgenic Arabidopsis.

(a) Sequence comparison of target regions in UVR8 after CRISPR/Cas9-mediated gene editing. Dashes indicate deletions. (b) Comparison of *UVR8* expression levels in transgenic Arabidopsis with WT by RT-qPCR analysis. The data are presented as means (*n* = 3). Error bars show SD derived from three biological replicates. (c) The expression of *HY5* in Arabidopsis plants irradiated by 280 nm UV-LED at 0.5, 2.5, and 4.5 µmol m^{-2} s^{-1} was analyzed by RT-qPCR. WT without irradiation and dark storage was used as a control to compare the expression levels. The data are presented as means (*n* = 3). Error bars show SD derived from three biological replicates. Asterisks indicate significant differences using Welch's *t*-test (**P*-value < 0.05). (d) *HY5* (AT5G11260) sequence of *uvr8* mutant line 4-38. The primers used are shown in the Supplementary Table S15. WT, wild type; *UVR8*, *UVR8* overexpression line 11-6; *uvr8*, *uvr8* mutant line 4-38.
